# Spatial distribution and determinants of bottle feeding among children 0-23 months in Ethiopia: spatial and multi-level analysis based on 2016 EDHS

**DOI:** 10.1186/s12887-022-03181-w

**Published:** 2022-03-09

**Authors:** Daniel Gashaneh Belay, Mihret Getnet, Yonas Akalu, Mengistie Diress, Yibeltal Yismaw Gela, Amare Belete Getahun, Desalegn Anmut Bitew, Bewuketu Terefe, Yitayeh Belsti

**Affiliations:** 1 Department of Human Anatomy, College of Medicine and Health Sciences, University of Gondar, Gondar, Ethiopia; 2 Department of Epidemiology and Biostatistics, Institute of Public Health, College of Medicine and Health Sciences, University of Gondar, Gondar, Ethiopia; 3 Department of Human Physiology, School of Medicine, College of Medicine and Health Sciences, University of Gondar, Gondar, Ethiopia; 4 Department of Anesthesia, School of Medicine, College of Medicine and Health Sciences, University of Gondar, Gondar, Ethiopia; 5 Department of Reproductive Health, Institute of Public Health, College of Medicine and Health Sciences, University of Gondar, Gondar, Ethiopia; 6 Department of Community health nursing, School of Nursing, College of Medicine and Health Sciences, University of Gondar, Gondar, Ethiopia

**Keywords:** Bottle feeding, Spatial, Ethiopia

## Abstract

**Background:**

Bottle feeding is associated with diarrheal disease morbidity and mortality and risk of pyloric stenosis, especially in developing countries. Even though, World Health Organization (WHO) recommended avoiding bottle feeding among children, still higher magnitude was reported in developing countries. This study aimed to assess the spatial distribution and determinants of bottle feeding among children 0-23 months in Ethiopia.

**Methods:**

This study was conducted based on Ethiopian Demographic and Health Surveys data (EDHS). The data were weighted using sampling weight for probability sampling and non-response to restore the representativeness of the data and get valid statistical estimates. Then a total of 4,275 weighted samples of under two years children were used to investigate the study. The data were cleaned using MS excel and extracted and analyzed using STATA V.16 software. A multilevel binary logistic regression model was fitted. P-value < 0.05 was taken to declare statistical significance. A spatial analysis was done using ArcGIS and SaTScan software.

**Results:**

The prevalence of bottle feeding practice among under two years children in Ethiopia were 13.5% (95%CI: 11.16, 15.29) and ranges from the lowest 5.16% (95% CI: 3.28, 78.73) Amhara region to the highest 55.98% (95% CI: 47.98, 61.46) Addis Ababa region. Women with secondary and above education status [AOR=2.49; 95%CI; 1.66, 3.74], women from richest household [AOR=1.33; 95%CI; 1.01, 1.78], child 12-23 months age [AOR= 1.59; 95%CI; 1.23, 2.05], multiple birth [AOR=4.30; 95%CI; 1.88, 9.84], rural residence [AOR=0.49; 95%CI; 0.16, 0.82] and large central region [AOR= 0.15; 95%CI; 0.08, 0.27] have significantly associated with bottle feeding. Addis Ababa, Central Oromia, Dire Dewa, Somali and Harari regions were the hot spot areas for bottle feeding practice among under two years children.

**Conclusion and recommendations:**

The prevalence of bottle feeding practices in Ethiopia is relatively moderate. Maternal education, wealth index, child age, multiple births, residence and region were significant predictors of bottle feeding. These findings highlight that, the Ministry of Health Ethiopia (MOH), policymakers, and other stakeholders had better give prior attention to preventable factors such as empowering women, enhancing household wealth status to decreasing bottle feeding practice in Ethiopia.

## Background

Infant and young child feeding (IYCF) practices determine under two years children's nutritional status and eventually impact on child survival, growth, and development [1]. In developing countries such as Ethiopia with high rates of infectious diseases, and malnutrition, as result morbidity and mortality of children, appropriate infant feeding has important implications for immediate and future health [1, 2]. The World Health Organization (WHO) recommendations of age-appropriate breastfeeding for under two years children states that, children aged 0-5 months should in exclusively breastfed and children aged 6-23 months should receive both breast milk and complementary food [3, 4].

Despite the recommendations of breastfeeding, in developing countries the prevalence and duration of breastfeeding are declining rapidly and being replaced by bottle feeding [5]. Bottle feeding is the practice of feeding an infant any liquid (including breast milk) or semi-solid food from a bottle with a nipple/teat [6, 7]. It is one of seven optional indicators for the assessment of IYCF practice launched by WHO [4].

Bottle feeding is not a recommended type of feeding system for children as it can lead to an increased incidence of excessive weight gain, malnutrition, iron depletion, and decreased birth spacing [8]. Even the expressed breast milk could increase infant weight gain if it is fed by the bottle [8]. Escaping pacifiers or artificial teats is a crucial strategy to promote universal breastfeeding [9]. Exposure of infants to bottle feeding has been strongly associated with poor breastfeeding conditions [7, 9]. This might be confusion of nipple occurs when infants are exposed to bottle and breastfeeding methods, resulting in the infant refusing to breastfeed [2, 9]. Furthermore, feeding bottles have been linked to diarrheal disease morbidity and death as it is difficult to keep it clean especially in developing countries where sanitation is poor [2, 10]. Bottles with a nipple are particularly more prone to contamination [4] and increase the prevalence of dental carries among children [11]. Studies also showed that, bottle-fed infants experienced a 4.6 fold higher risk of pyloric stenosis [12].

The prevalence of bottle feeding among under two years children was 35.7% in Namibia [13] and 12 % in Ghana [14]. A higher prevalence (38%) was reported in the Oromia region of Ethiopia [15], and 19.6% in Holeta town, Central Ethiopia [2].

The principal reasons of mothers for having bottle feeding of their child were due to insufficient breast milk [16], easiness for feeding of the child [2], to stop crying of the child [17], and to promotes growth of children [18]. The age of mothers and children [2], and getting advice/counseling on risks of bottle feeding had also significant associations with bottle feeding practice [2].

Even though child nutrition practice has several interwoven factors which operate concurrently and mostly depend on socio-economic status of the population [1, 19–22], no study assesses the community and individual level determinants of bottle-feeding simultaneously and its spatial distribution in Ethiopia. Therefore this study aimed to assess the spatial distribution and individual level and community level factors associated with bottle feeding among children 0-23 months in Ethiopia.

## Methods

### Study setting, and period

The fourth Ethiopian Demographic and Health Survey (EDHS, 2016) was conducted from January 18, 2016, to June 27, 2016 [23]. Ethiopia is an East African country (3^0^ -14^0^ N and 33^0^ – 48^0^E) with 1.1 million Sq. km coverage and the second most populous country in Africa with an estimated population of 100,613,986. Administratively, Ethiopia is federally decentralized into nine regions and two city administrations. Kebele is the lowest administrative unit [24].

### Source and study population

The source population was all children aged 0-23 months preceding five years of the survey period in Ethiopia. Whereas, the study population was children aged 0-23 months preceding five years the survey period in the selected Enumeration Areas (EAs). Mothers who had more than one child within the two years preceding the survey were asked questions about the most recent child. But mothers who had twin birth for the last birth were asked for both children [25].

All children aged 0-23 months preceding five years of the survey in the selected EAs in each country were included in this study. Recent birth children who were died were excluded from the study. Based on DHS recode manual for the management of handling of missing value, missing and “don’t know” responses on whether drank from a bottle with a nipple yesterday during the day or night are included in the study but considered as not used bottle feeding [26]. Finally, the unweighted 4,054 samples were used for analysis in this study from EDHS, 2016.

### Sampling technique

The EDHS used a stratified two-stage cluster sampling technique using the 2007 Population and Housing Census (PHC) as a sampling frame. Stratification was achieved by separating every eleven regions into urban and rural areas. In total, 21 sampling strata have been created. Enumeration Areas (EAs) were the sampling units for the first stage of sampling. Therefore, in the first stage, 645 EAs (202 in the urban area and 443 in the rural) were selected with probability selection proportional to the EA size and independent selection in each sampling stratum. In selected EAs, households (HHs) comprise the second stage of sampling. After the listing of the households, in the second stage, on average, 28 households have been systematically selected [26]. The detailed sampling procedure was available in each DHS reports from the Measure DHS website [24].

### Study variables

The outcome variable of this study was having bottle feeding practice of children 0-23 months. During the survey, their mother was asked questions about their under 2 years children who drank from a bottle with a nipple yesterday during the day or night before the interview [24].

Individual and community-level independent variables have been studied. The individual-level factors include socio-demographic characteristics such as; the age of the mother, mother employment, marital status, family size, maternal education, media exposure, and household wealth status were included. Child-related factors such as the age of the child, sex of the child, the plurality of birth, and are all taken into account. Health service utilization-related factors such as place of delivery, pregnancy wontedness, and ANC visit were also considered. The community-level factors include; distance from health facilities, place of residence, and region were considered.

Media exposure is created from three variables; reading newspapers, watching TV, and listening to the radio. If a woman has exposure to at least one type of media, she is considered exposed to media [27]. Based on the development status and the need for governmental support, the 11 regions of Ethiopia are categorized into three groups; ‘three Metropolis’ (Addis Ababa, Harari, and Diredewa), large central (Tigray, Amhara, Oromia, SNNPR ), and “small peripherals” (Afar, Benishangul Gumuz, Gambelia and Somali) [27].

### Data source, processing, and analysis

The data source for this study was the recent standard Demographic and Health Survey (DHS) data obtained from the official DHS measure after permission has been obtained via an online request by specifying the objectives of this study. The standard DHS data set was used to get all parameters and a large sample size which can be representative of the source [24]. The kid’s records (KR) DHS datasets were downloaded in STATA format. After the data was accessed, it was cleaned, coded, and merged to produce favorable variables for the analysis. Then before any statistical analysis, the data were weighted using sampling weight for probability sampling and non-response to restore the representativeness. Finally, a total weighted sample of 4, 275 children in the age category of 0-23 months were included in this study from EDHS, 2016. Microsoft Excel and STATA 16 software were used to generate both descriptive and analytic statistics to describe variables in the study using statistical measurements.

### Model building for multi-level analysis

Since DHS data has hierarchical nature which means children aged 0-23 months were nested within-cluster, the standard logistic regression model assumptions might be violated. Therefore, a multilevel binary logistic regression which includes four models was fitted. The first was the null model which was used to assess the variability of bottle feeding across the cluster. The second model includes individual-level variables whereas the third model includes community-level variables. Both individual and community level variables were fitted simultaneously with the prevalence of bottle feeding in the last model (Model 4). The log likely hood and deviance test were used for model comparisons and the model with the highest log likely hood and the lowest deviance value was selected as the best fitted model. The variance inflation factor (VIF) was used to detect multicollinearity, and all variables had VIF values less than 10 and the mean VIF value of the final model was 1.50.

### Parameter estimation method

The generalized linear mixed model (GLMM) model in which the linear predictor contains both random and fixed effect analyses were used for this study. In the fixed effects measure of association, factors with a *p*-value ≤ 0.2 have been selected as candidates for the final model. Associations between bottle feeding and independent variables were assessed, the strength was presented using adjusted odds ratios and 95% confidence intervals with a *p*-value of <0.05 [28–30].

$$Log\ \left(\frac{\pi ij}{1-\pi ij}\right)=\beta o+\beta 1 xij+\beta 2 xij+\dots uj+ eij$$

Where, *πij*: the probability of having bottle feeding, 1 − *πij*: the probability of having bottle feeding, *β*1xij are individual and community level variables for the i^th^ individual in group j, respectively. The ß’s are fixed coefficients indicating a unit increase in X can cause a ß unit increase in the probability of bottle feeding. While the ß0 is intercept that is the effect on bottle feeding when the effect of all explanatory variables is absent. The uj shows the random effect (effect of the community on the mother’s decision to provide bottle feeding) for the j^th^ community. The clustered data nature and the within and between community variations were taken into account assuming each community has a different intercept (β0) and fixed coefficient (β) [28, 30, 31].

The measure of variation or random effects were estimated by the median odds ratio (MOR), Intra Class Correlation Coefficient (ICC), and Proportional Change in Variance (PCV).

The MOR is defined as the median value of the odds ratio of bottle feeding between the area at the highest risk and the area at the lowest risk when randomly picking out two clusters.

MOR = exp.[√(2 × VA) × 0.6745], or $\mathrm{MOR}={e^{0.95}}^{\sqrt{VA}}$ where; VA is the area level variance [28–30].

The PCV reveals the variation in bottle feeding among children 0-23 months explained by factors. The PCV is calculated as; $PCV=\frac{Vnull- VA}{V\ null}\ast 100\%$ where; Vnull = variance of the initial model, and VA = variance of the model with more terms.

The ICC which reveals the variation of bottle feeding between clusters is calculated as;$\kern0.5em ICC=\frac{VA}{VA+3.29}\ast 100\%$, where; VA = area/cluster level variance [28–30].

### Spatial analysis of bottle feeding among 0-23 months children in Ethiopia

Global Moran’s I statistic spatial autocorrelation measure was used to assess the spatial distribution of bottle feeding among 0-23 months children in Ethiopia [32]. Getis-Ord Gi* statistic hot spot analysis was used to show significant hot spot or cold spot areas for bottle feeding among 0-23 months of children. The proportion of bottle-feeding among 0-23 months’ children in each cluster was taken as an input for hotspot analysis. To predict bottle feeding among 0-23 months children in Ethiopia for unsampled areas based on sampled clusters, a spherical semivariogram ordinary kriging type spatial interpolation technique was used. Bernoulli based model spatial scan statistics were employed to determine the geographical locations of statistically significant clusters for bottle feeding among 0-23 months children using Kuldorff’s SaTScan version 9.6 software [33]. The scanning window that moves across the study area in which children who had bottle feeding were taken as cases and those children who had not bottle feeding were taken as controls to fit the Bernoulli model.

## Results

### Socio-demographic characteristics of mothers or caregivers

Total weighted samples of 4,275 children of age 0-23 months were included in this study. Half (50.93%) of mothers of children were found in the age group of 20–35 years, with a median age of 28 (IQR: 9) years. Nearly three-fifths of women (60.31%) had no formal education. Eighty-five percent of the households were headed by men. Most (87.9%) of the respondents were rural inhabitants. Ninety percent (90%) of women included in the study were from large central regions (Table 1).

**Table 1:** Socio-demographic characteristics of the study mothers/caregivers in a study of trend and determinants of bottle feeding among less than two years children in Ethiopia: based on 2016 EDHS

Variables	Categories	Weighted Frequency (n)	Weighted Percentage (%)
Age of women (years)	15–19	1,251	29.27
	20–35	2,177	50.93
	36-49	846	19.8
Sex of household head	Male	3,695	86.44
	Female	580	13.56
Educational attainment of women	No education	2,578	60.31
	Primery education	1,314	30.74
	Secondary & above	382	8.95
Occupation of women	Not working	2,505	58.6
	Worked	1,770	41.4
Marital status of a mother	Married	4,026	94.17
	Not married	249	5.83
House hold family size	1-4	1,249	29.21
	5-10	2,911	68.1
	> 11	115	2.69
Media exposure	No	2,813	65.8
	Yes	1,462	34.2
Wealth index	Poorest	1,927	45.08
	Middle	907	21.22
	Richest	1,441	33.7
Residence	Urban	517	12.1
	Rural	3,758	87.9
Region	Metropolis	138	3.23
	Large central	3,860	90.29
	Small periphery	277	6.48

### Child related characteristics and health service utilization factors

From the total weighted sample of 4,275 children of age 0-23 months, nearly similar proportions of males (47.43%) and females (52.57%) were studied. Nearly half of (46.88%) the children were found in the age group from 12-23 months with a median age of 11 (IQR: 11) months. Almost all (97.66%) of childbirth were singleton. Moreover, only one-third of (36.77%) infants were delivered at the health facility (Table 2).

**Table 2:** Child related characteristics and health service utilization factors for trend and determinants of bottle feeding among less than two years children in Ethiopia: based on 2016 EDHS

Variables	Categories	Weighted Frequency(n)	Weighted Percentage (%)
Sex of child	Male	2,027	47.43
	Female	2,248	52.57
Age of child	0-5 months	1,404	32.85
	6-11 months	867	20.27
	12-23 months	2,004	46.88
Plurality	Single	4,175	97.66
	Multiple	100	2.34
Pregnancy wontedness	Wanted	3,139	73.43
	Unwanted	1,136	26.57
ANC visits	No ANC	1,441	34.72
	At least one ANC	2,710	65.28
Place of delivery	Home delivery	2,703	63.23
	Health facilities	1,572	36.77
Distance from health facilities	Not big problem	1,688	39.47
	Big problem	2,587	60.53

### Prevalence of bottle feeding among children less than two years in Ethiopia

**Fig. 1:**
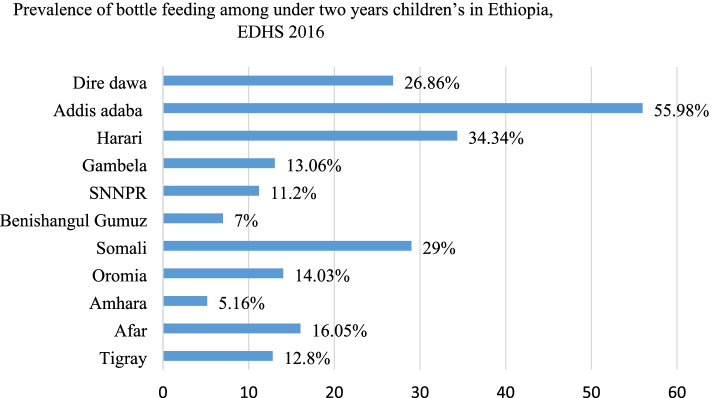
Regional prevalence of bottle feeding among children under two years in Ethiopia, 2016 EDHS

The overall prevalence of bottle feeding practice among children less than two years in Ethiopia was 13.05% (95% CI: 11.16, 15.29). The highest prevalence was seen in Addis Ababa 55.98% (95% CI: 47.98, 61.46) and the lowest prevalence was seen in the Amhara region 5.16% (95% CI: 3.28, 78.73) (Fig. 1). Bottle feeding practice was more common among children with 6-11 months (19.20%), but it was 10.45% in children with 0-5 months and 13.17% in children with 12-23 months.

### Multilevel model parameter results

#### Random effect and model comparison

The ICC value in the null model was showed 32% of the variations in bottle feeding practice among children aged 0–23 months were due to cluster differences. The MOR in the null model, also revealed that the median odds ratio between the higher and lower risk area of bottle feeding among clusters was 3.35. Moreover, about 32% of the variation in bottle feeding 0-23 children were explained by the final model (model four). Likelihood and deviance were used for model comparison and the model with the highest likelihood and the lowest deviance (model 4) were considered as the best fit model. There was no multicollinearity between independent variables in all models based on the Variance Inflation Factors (VIF) results (Table 3).

**Table 3:** Parameters and model fit statistics for multilevel regression analysis models

Parameters	Null model	Model 2	Model 3	Model 4
Cluster level Variance	1.609	1.357	1.046	1.0897
ICC	0.32	0.29	0.24	0.25
MOR	3.35[3.24, 3.43]**	3.03	2.64	2.69
PCV	Reference	0.16	0.35	0.32
Model fitness				
Likelihoods	-10,074	-5,776	-6,084	-5,636
Deviance	5,037	2,888	3,042	2,818
Mean VIF	---	1.32	1.87	1.50

*ICC* Inter cluster correlation coefficient , *MOR* Median odds ratio, *PCV* proportional change in variance, *VIF* Variance Inflation Factors

* = *P*-value < 0.05. ** = *P* value < 0.01. *** = *P* value < 0.001

### Multi-level analysis of determinant of bottle feeding among children age 0-23 Ethiopia

Both individual and community level variables which had a p-value<0.20 in the bivariable analysis were eligible for multivariable analysis. Since model four had the lowest deviance and was selected as the better fitted model, variables such as the educated status of women, age of the child, the plurality of birth, and region were significant variables in this model.

Primary and above primary educated women were 1.49 and 2.49 times more likely to have bottle feeding taken children than women with no formal education [AOR=1.49; 95%CI; 1.14, 1.94] and [AOR=2.49; 95%CI; 1.66,3.74] respectively. The odds of having bottle feeding among children from rich wealth status families were 1.33 times higher as compared to a child from poor wealth families [AOR=1.33; 95%CI; 1.01, 1.78].

Children whose age found 6-11 months and 12-23 months, were 2.36 and 1.59 times more likely to have bottle feeding as compared to a child with 0-5 months of age [AOR= 2.36; 95%CI; 1.77, 3.14] and [AOR= 1.59; 95%CI; 1.23, 2.05] respectively. Being multiple births were 4.3 times more likely to have bottle feeding than those who were singleton [AOR=4.30; 95%CI; 1.88, 9.84].

Children who were living in rural residences were 51% less likely to have bottle feeding practice than urban residents [AOR=0.49; 95%CI; 0.16, 0.82]. Children who live in large central and small periphery regions were 85% and 50% less likely to have bottle feeding than those who live in metropolis cities (Addis Ababa, Dire Dawa, and Harari) [AOR= 0.15; 95%CI; 0.08,0.27] and [AOR= 0.50; 95%CI; 0.26, 0.99] respectively (Table 4).

**Table 4:** Multilevel analysis of factors associated with bottle feeding among children age 0–23 months in Ethiopia, EDHS 2016

Variables	Categories	^a^Model 2	Model 3	Model 4
		AOR [95% CI]	AOR [95% CI]	AOR [95% CI]
Age of women (years)	15–19	1.00	**------------**	1.00
	20–35	1.01[0.78, 1.33]	**------------**	0.97 [0.74, 1.27]
	36-49	0.89 [0.62, 1.28]	**------------**	0.86 [0.60,1.25]
Sex of household head	Male	1.00	**------------**	1.00
	Female	1.12 [0.82, 1.52]	**------------**	0.99 [0.73, 1.36]
Educational attainment of women	No education	1.00	**------------**	1.00
	Primery education	1.49 [1.14, 1.94]*	**------------**	1.49[1.14, 1.94]*
	Secondary&above	3.07 [2.07, 4.55]**	**------------**	2.49 [1.66,3.74]***
Occupation of women	Not worked	1.00	**------------**	1.00
	Worked	0.91[0.73, 1.14]	**------------**	0.95[0.76,1.19]
Marital status of a mother	Married	1.00	**------------**	1.00
	Not married	1.07[0.67, 1.69]	**------------**	1.07[0.67,1.70]
Household family size	1-4	1.00	**------------**	1.00
	5-10	0.78 [0.60, 1.01]	**------------**	0.80[0.62,1.03]
	>11	1.57[0.85, 2.89]	**------------**	1.62 [0.88,2.97]
Media exposure	No	1.00	**------------**	1.00
	Yes	1.06 [0.87, 1.37]	**------------**	1.01 [0.77, 1.31]
Wealth index	Poorest	1.00	**------------**	1.00
	Middle	0.89 [0.65, 1.23]	**------------**	0.99 [0.72, 1.36]
	Richest	1.34 [1.06, 1.79]*	**------------**	1.33 [1.01, 1.78]*
Sex of child	Male	1.00	**------------**	1.00
	Female	0.80 [0.65, 0.98]*	**------------**	0.81 [0.66, 1.00]
Age of child	0-5	1.00	**------------**	1.00
	6-11	2.41 [1.81, 3.21]***	**-----------**	2.36 [1.77, 3.14]***
	12-23	1.63 [1.26, 2.10]***	**-----------**	1.59 [1.23, 2.05]***
Plurality of birth	Single	1.00	**------------**	1.00
	Multiple	4.10 [1.78, 9.48]**	**------------**	4.30 [1.88, 9.84]***
Pregnancy wontedness	Wanted	1.00	**------------**	1.00
	Unwanted	0.77 [0.59, 0.99]*	**------------**	0.80 [0.62, 1.04]
ANC visits	No ANC	1.00	**------------**	1.00
	At least one ANC	0.93 [0.72, 1.21]	**------------**	0.93 [0.72, 1.20]
Place of delivery	Home delivery	1.00	**------------**	1.00
	Health facilities	1.19 [0.90, 1.58]	**------------**	1.11 [0.83, 1.48]
Community level variables				
Distance from health facilities	Not big problem	**------------**	1.00	1.00
	Big problem	**------------**	**0.96 [0.76, 1.21]**	1.08 [0.83, 1.39]
Residence	Urban	**------------**	1.00	1.00
	Rural	**------------**	0.52[0.34, 0.80]*	0.49 [0.16, 0.82]*
Region	Metropolis	**------------**	1.00	1.00
	Large central	**------------**	0.15 [0.08, 0.259]**	0.15 [0.08,0.27]*
	Small periphery	**------------**	0.41 [0.26, 0.77]*	0.50 [0.26, 0.99]*

*AOR* Adjusted Odds Ratio; *CI* Confidence Interval

* = *P*-value < 0.05, ** = *P* value < 0.01, *** = *P* value < 0.001

^a^Model 1(null model) = the model which contains only with dependent variable and values expressed

Spatial analysis of bottle feeding among 0-23 months children in Ethiopia: based on 2016 EDHS

**Fig. 2:**
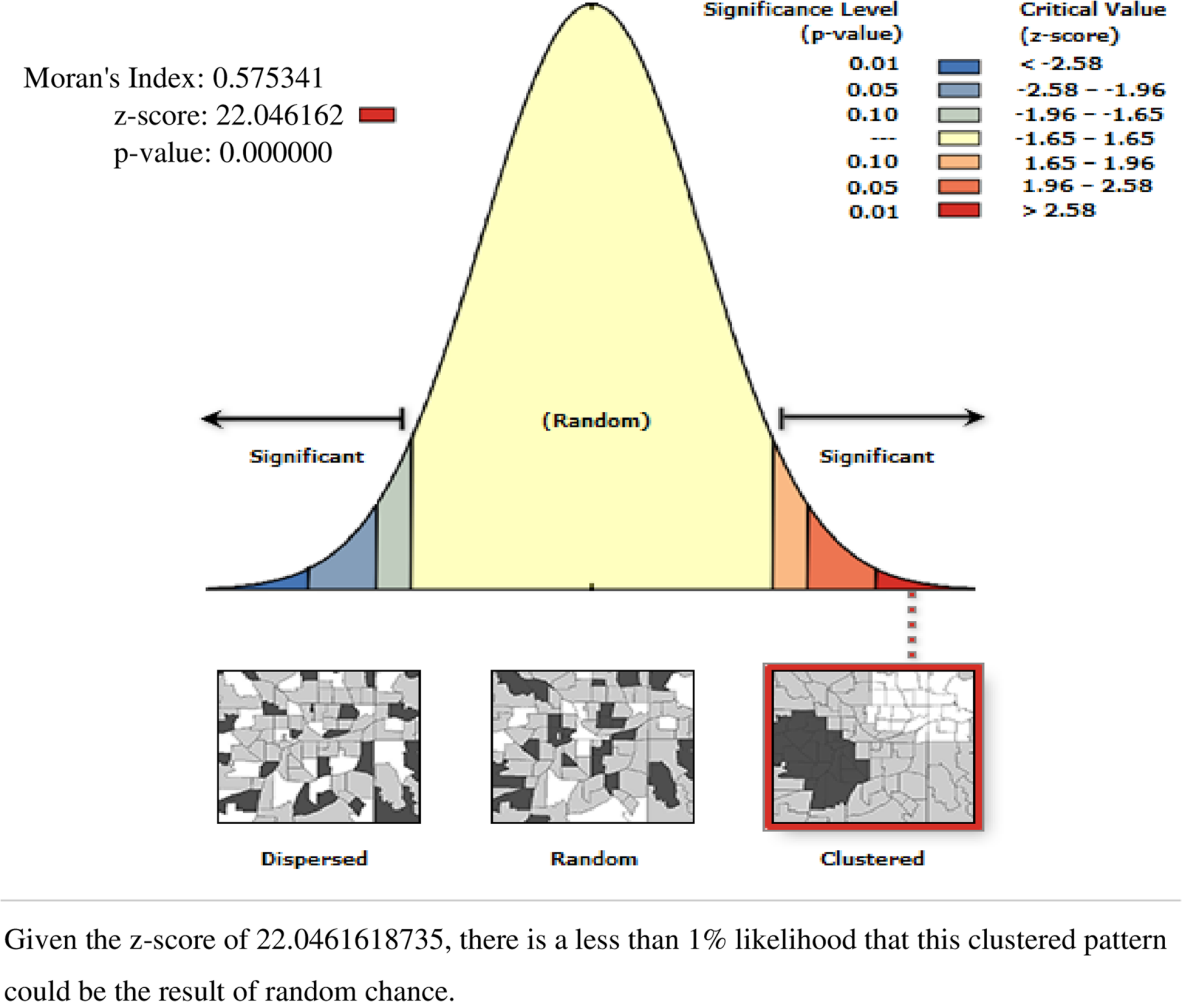
Spatial autocorrelation analysis of bottle feeding among 0-23 months children in Ethiopia, 2016 EDHS

**Fig. 3:**
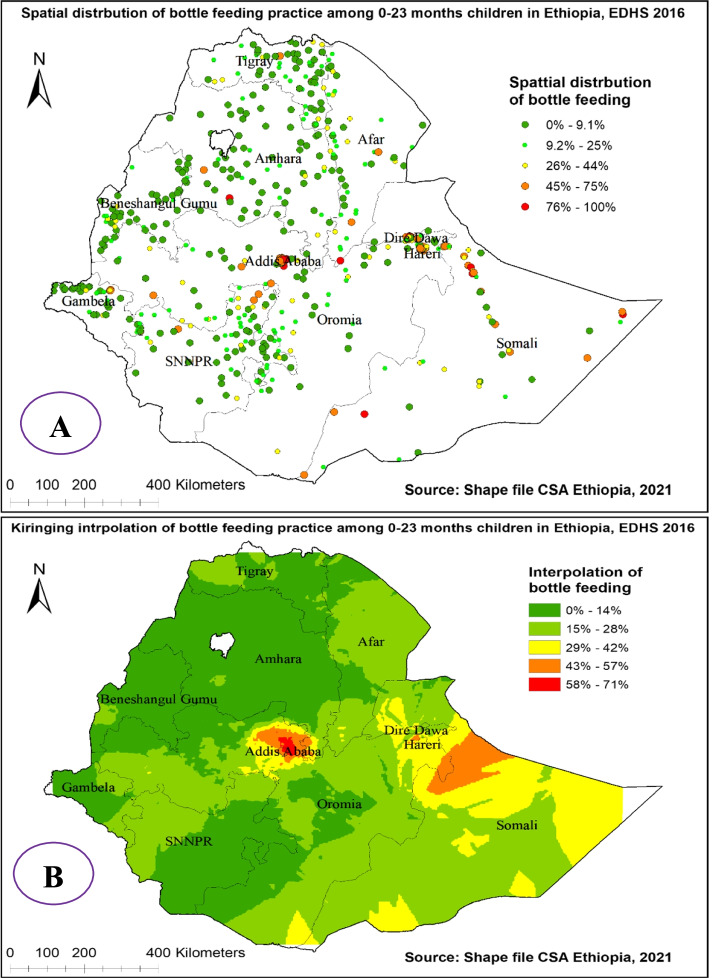
Spatial distribution (**A**) and kriging interpolation (**B**) of bottle feeding among 0-23 months children in Ethiopia, 2016 EDHS

The spatial distribution of bottle feeding practice among 0-23 months children in Ethiopia showed significant spatial variation over regions in the country, which is found to be clustered with Global Moran’s I value 0.575 with (*p*< 0.01) (Fig. 2). It is more common in Addis Abeba, Dire Dewa, and Harari regions and ranges from 75% to 100% (Fig. 3 (A)).

The Kriging interpolation methods of predicting bottle feeding among 0-23 months children in Ethiopia over the area was increased from green which indicates low- risk to red-colored which indicates high-risk areas. The prevalence of high-risk areas predicted bottle feeding among 0-23 months was moderate and ranges from 58% to 71% and located in Dire Dewa, Addis Ababa, and Somali regions. Whereas the lower predicted area was seen in, Amhara, Benishangul Gumuz, Gambelia, and SNNP (south nation nationalities and peoples of Ethiopia) regions and ranges from 0% to 14% (Fig. 3 (B)).

### Hot spot area and spatial window analysis of bottle feeding practice in Ethiopia

**Fig. 4:**
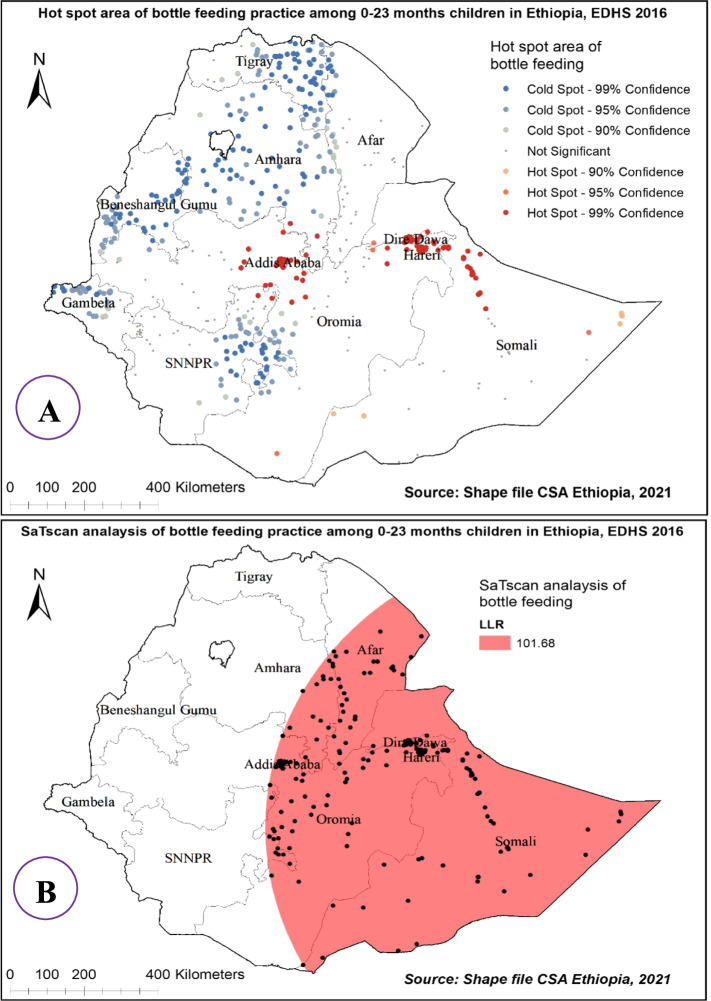
Hot and cold spot area (**A**), and Sat Scan analysis (**B**) of bottle feeding among 0-23 months children in Ethiopia, 2016 EDHS

The hot spot analysis of bottle feeding practice among 0-23 month’s children in 2016 EDHS showed that Addis Ababa, Central Oromia, Dire Dewa, Somali, and Harari regions were hot spot areas for bottle feeding practice whereas, Tigray, Amhara, Benishangul Gumuz, Gambelia and SNNP regions were cold spot areas (Fig. 4 (A)).

There were 283 primary clusters of bottle feeding practice among 0-23 month’s children in Ethiopia. These were located in the entire Somali, Dire Dawa, Harari, and Addis Ababa regions as well Eastern part of Afar, Amhara, and Oromia regions centered at 7.238405 N, 46.204502 E with an 863.79 km radius. Children which were found in the SaTScan window were 2.71 times more likely to use bottle feeding than out of window regions (RR=2.71, *P*-value<0.01) (Fig. 4 (B)).

## Discussions

The prevalence of bottle feeding practice among under two years children in this study was 13.5% (95% CI: 11.16, 15.29). This is in line with a study conducted in Ghana [14]. But lower than a study conducted in Indonesia [34], Pakistan [35], Eastern Sudan [36], and studies conducted in different districts of Ethiopia [2, 15, 37]. This difference might be due to the variations in socio-cultural aspects of the study participants regarding child feeding practices. Moreover having differences in knowledge of the study participants about the risks associated with bottle feeding might have a contribution to this difference.

The results of this study demonstrate that as the education status of the women increase, the use of bottle feeding of their child increases. This is in line with a study conducted in Indonesia [34] and a study in Namibia [13]. Time availability is a necessity for mothers to breastfeed their children. Since the educated mothers might have busy work schedule as compared to housewives (no workers), they may not have time to breastfeed [2, 34]. On the other hand, the result reflects that having awareness and understanding of the advantages of breastfeeding doesn’t depend on a mother’s educational status.

The current study found that the odds of having bottle feeding among children from a rich family were higher than a child from a poor wealth family. This is supported by a study conducted in Namibia [13], Pakistan [38], and Indonesia [34]. This might be due to that, rich family able to access breastfeeding alternatives, such as nipple or bottle feeding [34].

According to this study, as the age of the child increase, the chance of having bottle feeding increases. This is supported by a study conducted in Woldia Ethiopia [37], Holeta central Ethiopia [2], Namibia [13], Indonesia [34] which showed that children aged 6-23 months were more likely to use bottle feeding as compared to children aged 0-5 months. This is because, as child get older, they may have more feeding options, such as consumption of water, tea, and processed milk which may lead to a higher rate of bottle feeding [34]. On the other hand, children who are found aged 0-5 months had no well-matured gastrointestinal systems then bottle feeding during this age may be associated with problems in digestion and absorption, which eventually lead to diarrhea, vomiting, and infections. Then mothers who had such previous experience, may not start bottle feeding their child at an early age [37].

In this study, being multiple births were more likely to have bottle feeding as compared to singleton. This is in line with a study conducted in Japan [39]. Inadequate breastfeeding and competition for nutritional intake, occur more frequently in children of multiple births than in children of single births [40].

Children who were living in rural residents were less likely to have bottle feeding practices than urban residents in this study. This is comparable with a study conducted in Namibia [13], and in Indonesia [34] which shows children who lived in urban residence were more bottle feed than rural. This might be mothers in urban areas were more likely being higher socioeconomic status compared with those from rural areas and that could have made it easier to get information on breast milk and access to substitutes for breast milk [34, 38].

Based on the multilevel analysis of this study, children who lived in large central and small periphery regions were less likely to have bottle feeding than metropolis cities (Addis Ababa, Dire Dawa, and Harari). Moreover, the spatial analysis result also showed that bottle feeding practices among 0-23 month’s aged children in Ethiopia were not randomly distributed over regions in the country. It was more common in Addis Ababa, Dire Dewa, Harari, and Somali regions, whereas it was less distributed in Amhara, Benishangul Gumuz, and Tigray regions. This is supported by a study in Pakistan [38]. This might be due to that, most urban mothers are more likely to have paid employment, and the pressure to return to work after maternity leave might result in bottle usage [38].

Generally, the prevalence of bottle feeding practice in Ethiopia is relatively moderate as compared to other studies. Individual-level factors such as maternal education, wealth index, child age, and multiple births have a positive association with bottle feeding of the child. From community-level variables living in rural residences and living in the large central and small peripheral regions have a negative association with bottle feeding among children aged 0-23 in Ethiopia. To prevent or reduce the prevalence of bottle feeding among children aged 0-23 months in Ethiopia, the Ministry of Health Ethiopia (MOH), policymakers, and other stakeholders such as the ministry of education should work interactively. Promoting breastfeeding of children which targets children aged 6–23 months, multiple births, and rural residences and children from educated mothers.

### Strength and limitation

The main strength of this study was the use of the weighted nationally representative data with a large sample which makes it representative at country levels. Therefore, it has appropriate statistical power that can be generalized of the estimates in bottle feeding in the study setting to all children 0-23 during the study period. The spatial distribution is also useful. Since the data were collected cross-sectional by self-reported interview would be prone to recall and social desirability bias. The drawback of the secondary nature of data was inevitable.

## Data Availability

Data is available publically access from the open databases. It can be accessed by the following website.https://dhsprogram.com/data/dataset_admin/login_main.cfm?CFID=10818526&CFTOKEN=c131014a480fe56-4E0C6B7F-F551-E6B2-50
